# pH-dependent binding of guests in the cavity of a polyhedral coordination cage: reversible uptake and release of drug molecules†
†Electronic supplementary information (ESI) available: Experimental information; further details on the NMR titrations used to measure binding constants at different pH values; crystallographic data in CIF format (CCDC 1013340). See DOI: 10.1039/c4sc02090a



**DOI:** 10.1039/c4sc02090a

**Published:** 2014-07-31

**Authors:** William Cullen, Simon Turega, Christopher A. Hunter, Michael D. Ward

**Affiliations:** a Department of Chemistry , University of Sheffield , Sheffield S3 7HF , UK . Email: c.hunter@sheffield.ac.uk ; Email: m.d.ward@sheffield.ac.uk ; Fax: +44 114 2229346

## Abstract

A range of organic molecules with acidic or basic groups exhibit strong pH-dependent binding inside the cavity of a polyhedral coordination cage. Guest binding in aqueous solution is dominated by a hydrophobic contribution which is compensated by stronger solvation when the guests become cationic (by protonation) or anionic (by deprotonation). The Parkinson's drug 1-amino-adamantane (‘amantadine’) binds with an association constant of 10^4^ M^–1^ in the neutral form (pH greater than 11), but the stability of the complex is reduced by three orders of magnitude when the guest is protonated at lower pH. Monitoring the uptake of the guests into the cage cavity was facilitated by the large upfield shift for the ^1^H NMR signals of bound guests due to the paramagnetism of the host. Although the association constants are generally lower, guests of biological significance such as aspirin and nicotine show similar behaviour, with a substantial difference between neutral (strongly binding) and charged (weakly binding) forms, irrespective of the sign of the charged species. pH-dependent binding was observed for a range of guests with different functional groups (primary and tertiary amines, pyridine, imidazole and carboxylic acids), so that the pH-swing can be tuned anywhere in the range of 3.5–11. The structure of the adamantane-1-carboxylic acid complex was determined by X-ray crystallography: the oxygen atoms of the guest form CH···O hydrogen bonds with one of two equivalent pockets on the internal surface of the host. Reversible uptake and release of guests as a function of pH offers interesting possibilities in any application where controlled release of a molecule following an external stimulus is required.

## Abstract 2

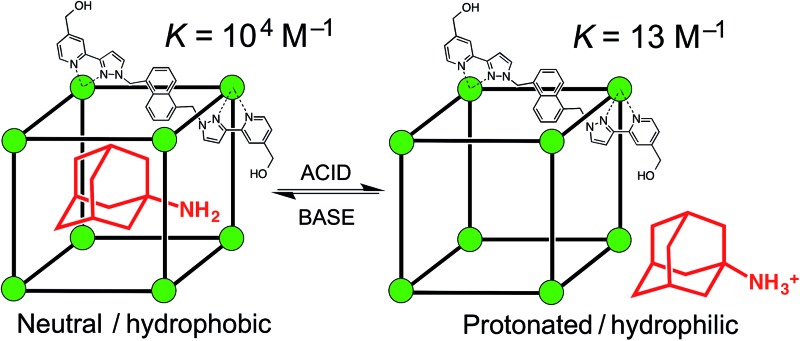
Binding of organic guests containing acidic or basic groups inside a water-soluble coordination cage host shows strong pH dependence.

## Introduction

A key goal of synthetic supramolecular chemistry is to control the use of weak, non-covalent interactions as the basis for planned self-assembly of combinations of molecular components with both *structures* and *functions* that are not accessible using conventional covalent-bond synthesis.^1^ One area which has seen huge progress in the last 20 years is that of the host–guest chemistry of hollow container molecules whose central cavity provides a tightly controlled microenvironment that is different from that of the bulk solvent and whose shape and size may be rigidly defined.^2,3^ These containers may be either organic capsules, often held together by hydrogen-bonding;^2^ or polyhedral coordination cages based on metal–ligand interactions.^3^

Within these classes of host there are now many well-characterised examples of guest binding with potential applications emerging in many areas where size/shape selective binding or transport of one specific guest can occur.^1–3^ These include catalysis; sensing; drug delivery; and stabilisation of reactive intermediates. The consequences of guest binding can include alteration of the conformation of flexible guests due to space restrictions;^4^ stabilisation of otherwise unstable molecules;^5^ and – at its most sophisticated – size- and shape-selective catalysis of reactions in the cavity.^6^

Despite the numerous examples of host–guest chemistry of container molecules, our ability to *control* guest uptake and release is limited. Interactions between host and guest cannot usually be altered which means that the affinity of the guest for the host is fixed, and movement into/out of the cavity is based on a simple equilibrium over which we can exert little control. A particular guest may be displaced from a cavity by adding a competing guest that binds strongly;^5a,7^ or concentrations of host and guest can be altered to adjust the position of an equilibrium without altering the equilibrium constant. In neither case is the strength of the interaction between host and guest modified.

It would be highly desirable therefore to find some external stimulus that can reversibly increase or decrease the affinity of a guest for its host, so that guest uptake and release can be triggered on demand. A few examples of such uptake/release do exist. Therrien *et al.* have reported a triangular cage complex which binds planar guest molecules and then moves through cell membranes whilst carrying the payload.^8^ Crowley *et al.* recently described a cage which binds two molecules of the drug *cis*-platin [*cis*-PtCl_2_(NH_3_)_2_] *via* H-bonding interactions: however removal of the guest (and, hence, delivery of the drug to its target) requires decomposition of the host cage.^9^ Clever *et al.* have prepared a cage in which (reversible) photoinduced rearrangement of the structure, which incorporates photochromic units in the ligands, resulted in the guest being ejected.^10^ Fujita *et al.* reported how a redox change of a ferrocene guest in a cage cavity – switching the guest between neutral and cationic forms – resulted in reversible uptake and release of the guests.^11^ Both Nitschke^12^ and Fujita^13^ have shown how simple basic guests (pyridine or *N*,*N*-dimethylaniline, respectively) are ejected from the cavity of a host cage host following protonation, allowing the use of a pH swing to control uptake/release in isolated cases. An interesting variant on this is the complete disassembly/re-assembly of a cage at different pH values which is associated with release/re-uptake of the guest.^14^ These existing examples can be conceptually separated into those that require rearrangement or decomposition of the *host cage* to liberate the guest,^8–10,14^ and those in which it is changing the charge on the *guest* that is the basis of uptake and release.^11–13^

This handful of disparate examples shows how the development of a mechanism for controlled uptake and release of guest molecules from containers according to an external stimulus is an important goal. A fully reversible uptake/release switching mechanism that can be applied to a wide range of guests under a wide range of conditions will make a major contribution to the development of useful functions from supramolecular assemblies in fields from medicine to catalysis.

**Fig. 1:**
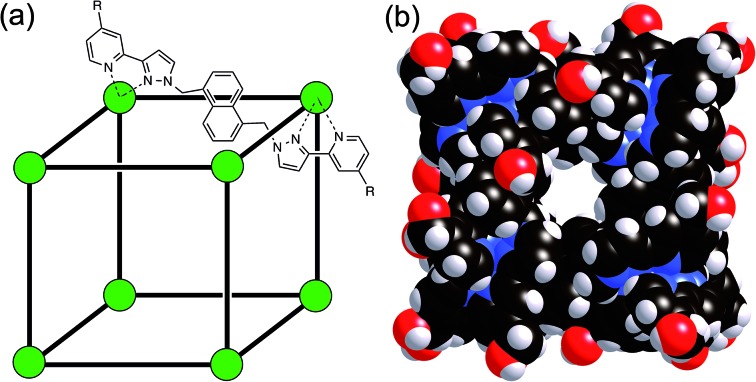
(a) Sketch of the cubic host cage showing the disposition of bridging ligands spanning each edge (R = CH_2_OH); (b) a space-filling view of the complete cage cation, showing the external O atoms of the hydroxyl groups in red (reproduced from ref. 16a).

We have recently reported the strongly size- and shape-selective host–guest chemistry of some octanuclear, approximately cubic, [Co_8_L_12_]^16+^ coordination cages.^15,16^ These cages contain a Co(ii) ion at each vertex and a ditopic bridging ligand L (containing two chelating pyrazolyl-pyridine termini) spanning each of the 12 edges (Fig. 1).^17^ In MeCN guest binding was dominated by interactions of the guests – which included a range of bicyclic organic species such as coumarin and isoquinoline-*N*-oxide – with the interior cavity walls. These interactions include a hydrogen-bonding interaction between the guests' exocyclic oxygen atom and a convergent set of weakly polar CH protons on the host, and also non-polar interactions between the guest and the cavity walls.^15^ In water however guest binding in the isostructural [Co_8_(L^w^)_12_]^16+^ cage (functionalised with hydroxy groups on the external surface, Fig. 1) is dominated by the hydrophobic effect. As long as the guest is not too large for the cavity, the binding affinity in water correlates very well with the total of the surface area of both host and guest that is desolvated when the hydrophobic surfaces come into contact. The strongest guest binding so far observed is with cycloundecanone, for which *K* > 10^6^ M^–1^.^16^

The importance of the hydrophobic effect^18^ in affording strong guest binding in this cage system^16^ has led us to examine binding of a wider range of hydrophobic guests. During the course of this work it became apparent that binding of guests with functional groups that can be protonated or deprotonated (pyridines, amines, imidazoles, carboxylic acids) showed a strong pH dependence. The use of pH changes to control supramolecular assemblies in many ways is well established.^19^ Prominent examples include changing the conformation of rotaxanes by protonation/deprotonation of specific sites on the axle;^20^ pH-induced disassembly of amphiphilic containers as a mechanism for drug release;^21^ and reversible control of the assembly/disassembly of crown ether/ammonium H-bonded systems using a pH swing.^22^

Accordingly we report here the results of a study showing how a pH swing can be used as the basis of reversible uptake/release of a range of guests, spanning a wide range of p*K*_a_ values, from the cavity of a coordination cage. Some of the guests have biological significance including use as prescription drugs.

## Results and discussion

Our recent work on binding of hydrophobic guests in the cavity of [Co_8_(L^w^)_12_]^16+^ (hereafter denoted **H**) in water showed that simple substituted adamantanes such as adamantanone and 1-acetyl-adamantane bound well (*K* > 10^4^ M^–1^), as the adamantyl group is a good size/shape match for the pseudo-spherical cavity of **H** in addition to having a high hydrophobic surface area.^16*b*^ As we reported before, the paramagnetism of [Co_8_(L^w^)_12_]^16+^, arising from the presence of high-spin Co(ii) ions, acts as a shift reagent which disperses the ^1^H NMR signals over a range of *ca.* 200 ppm.^15–17^ This makes it easy to separate the signals for free cage and the cage/guest complex under slow-exchange conditions, and integration of these signals at known concentrations of host and guest provides the association constants.

**Fig. 2:**
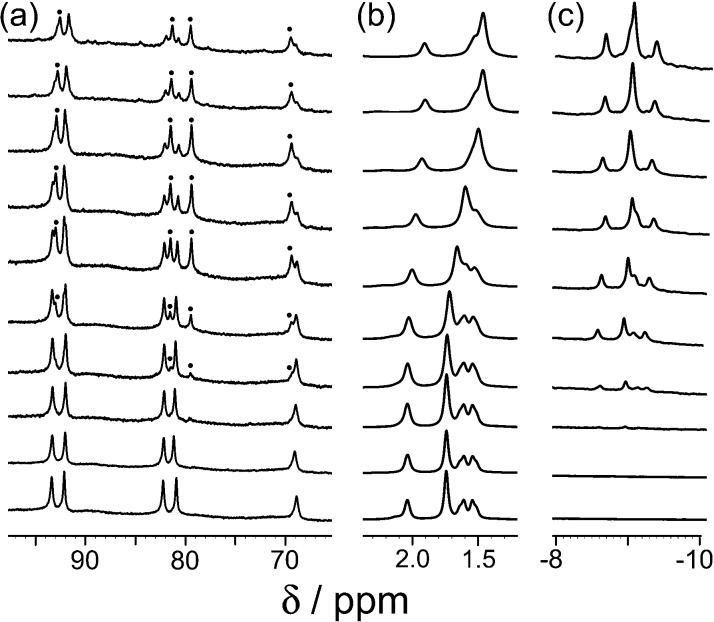
Series of partial ^1^H NMR spectra recorded for a mixture of host cage **H** (0.2 mM) and 1-aminoadamantane in D_2_O (1.26 mM), at pH values from 3.82 (bottom) to 12.46 (top). Progressing upwards the spectra show how 1-aminoadamantane enters the cavity as it is converted from the protonated to the neutral form at higher pH values; the signals marked • in part (a) are from the host–guest complex. pH values (from bottom up) are 3.82, 7.61, 8.62, 9.42, 9.98, 10.54, 10.90, 11.68, 12.01, 12.46.

We extended the search to other substituted adamantanes, and were initially surprised to find no evidence for binding of 1-aminoadamantane. On addition of an excess of guest to a sample of **H** in D_2_O, there was no change in the ^1^H NMR spectrum of the cage under the same conditions that showed strong binding for adamantanone and 1-acetyladamantane. On reflection, it seemed possible that this could be because this guest (p*K*_a_ = 10.9 for the protonated form) is protonated under neutral conditions. Protonation renders the guest more hydrophilic than the neutral form and could also result in electrostatic destabilisation of the complex, because the cage has a charge of 16+. To test this hypothesis, we performed a pH titration in an NMR tube containing fixed amounts of **H** (0.2 mM) and excess 1-aminoadamantane (1.26 mM) in D_2_O. Addition of NaOD or DCl allowed us to vary the pH over the range 4–12. Fig. 2 shows the evolution of three different regions of the ^1^H NMR spectrum as a function of pH. In the 70–95 ppm region [Fig. 2, column (a)], the signals due to the free host slowly decrease in intensity and are replaced by a new set of signals – always close to the parent signals – which are due to the host–guest complex. In some parts of the spectrum (*e.g.* the two signals at around 80 ppm), the separation between the signals due to free and bound host is sufficient to allow them to be integrated separately. In the 1–2 ppm region [column (b)], the signals due to protonated free guest move to a lower chemical shift as the pH increases, and the protonated 1-aminoadamantane cation becomes deprotonated. Finally, in the region between –8 and –10 ppm [column (c)], the signals due to bound guest have a negative chemical shift because of the proximity to the eight paramagnetic metal ions surrounding the cavity. At pH 7 there is no measurable bound guest; as the pH is raised, the amount of bound guest steadily increases as the free 1-amino-adamantane cation is deprotonated, and the neutral form enters the host cavity.

**Fig. 3:**
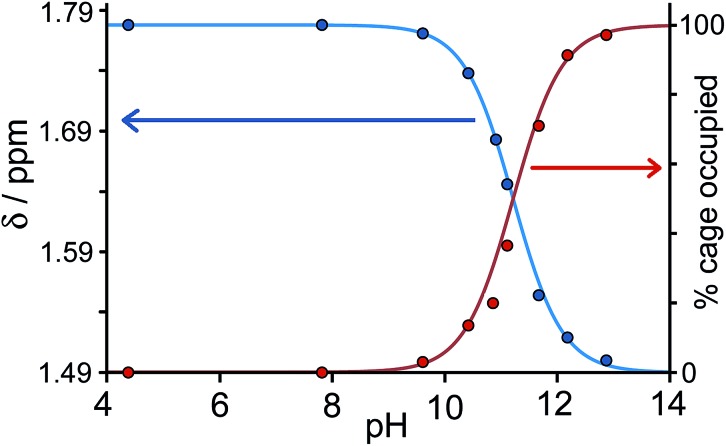
Graphical representation of data extracted from Fig. 2: (a) blue curve, chemical shift of one of the signals of 1-aminoadamantane as a function of pH; (b) red curve, occupancy of the cavity of **H** as a function of pH on the basis of ^1^H NMR signal integrals. The two curves mirror each other and intersect at the p*K*_a_ of 1-aminoadamantane.

Fig. 3 shows a summary of data extracted from the pH titration, *viz.* the proportion of cage occupied as a function of pH (in red), and the chemical shift of the most intense 1-aminoadamantane signal as a function of pH (in blue, *i.e.* a pH titration curve for the free guest).

It is clear that under these conditions – *i.e.* in the presence of excess guest – occupancy of the cavity goes from negligible to complete (within the limits of error of the NMR measurements), and the pH at which this process is 50% complete is the same as the p*K*_a_ of 1-aminoadamantane. In other words, the uptake and release of the guest from the cage cavity is driven by the deprotonation/protonation of the guest. Integration of the signals due to free and bound host at different concentrations of guest, during separate NMR titrations of **H** with 1-aminoadamantane at fixed pH values of 7 (weak binding limit) and 12 (strong binding limit), gave a binding constant of 1.0(3) × 10^4^ M^–1^ for neutral 1-aminoadamantane and 13(7) M^–1^ for the protonated form, which is a change of three orders of magnitude and corresponds to a difference of 17 kJ mol^–1^ between the binding free energies of the neutral and cationic forms. The process is fully reversible.

**Fig. 4:**
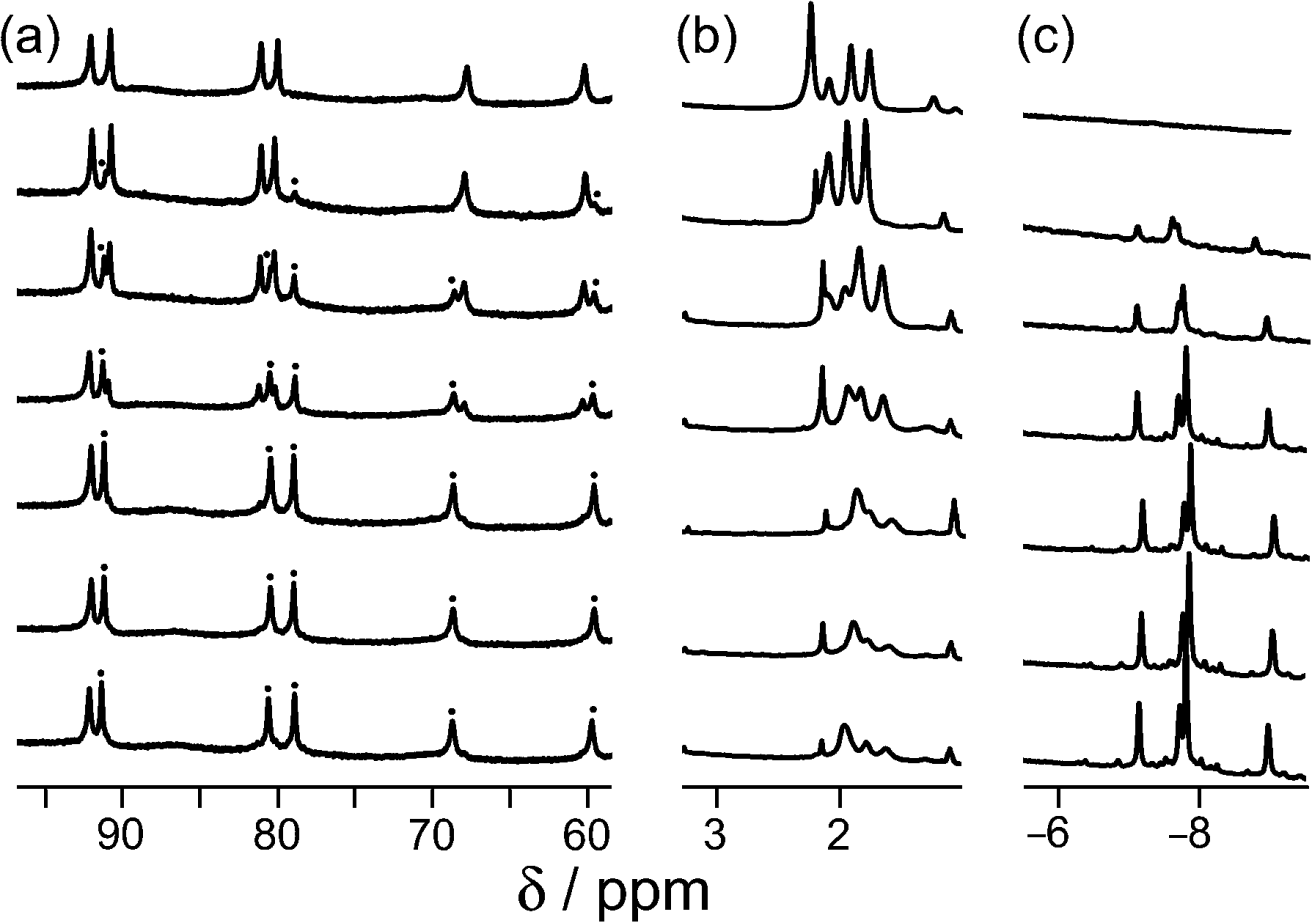
Series of partial ^1^H NMR spectra recorded for a mixture of host cage **H** and 1-adamantane-carboxylic acid in D_2_O (0.24 mM), at pH values from 2.31 (bottom) to 9.57 (top). Progressing upwards the spectra show how 1-adamantane-carboxylic acid is ejected from the cavity as it is converted from the neutral to the anionic form at higher pH values; the signals marked • in part (a) are from the host–guest complex. pH values (from bottom up) are 2.31, 4.79, 5.43, 6.34, 7.00, 8.02, 9.57.

**Fig. 5:**
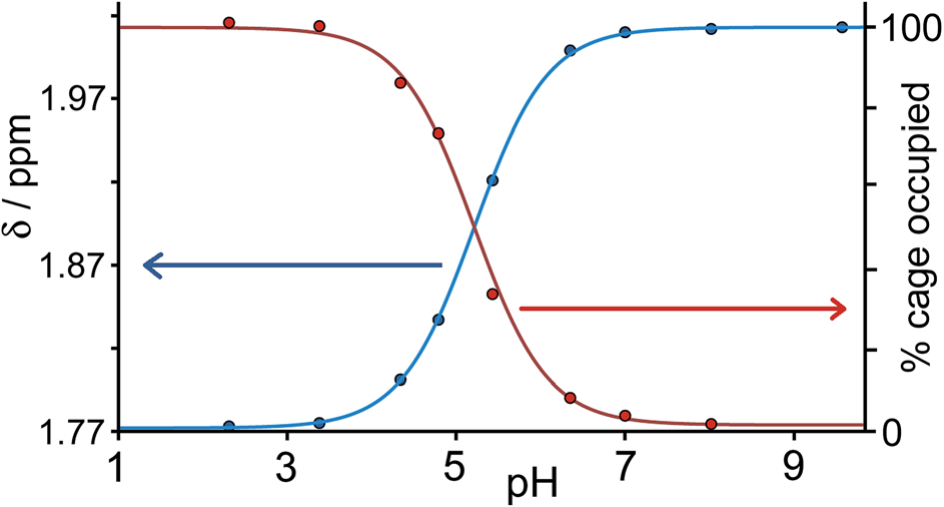
Graphical representation of data extracted from Fig. 4: (a) blue curve, chemical shift of one of the signals of 1-adamantane-carboxylic acid as a function of pH; (b) red curve, occupancy of the cavity of **H** as a function of pH on the basis of ^1^H NMR integrals. The two curves mirror each other and intersect at the p*K*_a_ of 1-adamantane-carboxylic acid.

Continuing with substituted adamantanes, we next examined 1-adamantane-carboxylic acid, which has a p*K*_a_ of 5.1, so the pH at which the host–guest interaction is switched on/off should be different. At pH values <5 there is a slight drift in the ^1^H NMR signals from the cage as a function of pH, due to deprotonation of some of the 24 externally-directed hydroxyl groups which are relatively acidic due to the high positive charge on the cage. However, binding of 1-adamantane-carboxylic acid is again in slow exchange on the ^1^H NMR timescale, so discrete signals were observed for free and bound host, and these were integrated to obtain the association constants for the neutral and deprotonated forms of the guest (Fig. 4). Again, the protonation/deprotonation equilibrium of the free guest was monitored by changes in the chemical shift of the signals due to the adamantyl protons around 2 ppm [Fig. 4, column (b)], and movement of the neutral form of the guest into the cavity at lower pH values is shown by the increasing intensity of the paramagnetically-shifted signals at around –8 ppm for the bound guest [column (c)]. Fig. 5 shows a graphical summary of both cavity occupancy and free guest ^1^H NMR chemical shift as a function of pH, showing again how the two curves mirror each other and intersect at the p*K*_a_ of 1-adamantane-carboxylic acid.

1-Adamantane-carboxylic acid binds two orders of magnitude more strongly in its neutral state [*K* = 8.2(2) × 10^4^ M^–1^], than in its anionic state [*K* = 9.0(5) × 10^2^ M^–1^]. This is the converse of what might be expected on purely electrostatic grounds given the positive charge of the cage, which means that any electrostatic interaction between host and guest is less significant than solvation effects. The carboxylate anion is bound more weakly simply because it is more hydrophilic and better solvated in water than the neutral carboxylic acid. To test this hypothesis, we measured the association constants for 1,3-adamantane-dicarboxylic acid over the same pH range. For the neutral diacid form at pH 3, *K* = 2.3(4) × 10^5^ M^–1^, whereas for the dianionic form at pH 8, the association constant was too low to measure at the accessible concentrations (*K* < 300 M^–1^).‡
‡The two p*K*_a_ values of adamantane-1,3-dicarboxylic acid were measured by ^1^H NMR pH titrations as 4.8 and 5.9, so we can be confident that the binding constants measured at pH 3 and 8 correspond to the neutral and dianionic forms of the guest, respectively. High concentrations of the dianion at pH 8 resulted in decomposition of the cage, limiting our estimate of the binding constant of the dianionic form to <300 M^–1^. For 1-adamantane-carboxylic acid, deprotonation results in a loss of binding free energy (ΔΔ*G*) of 11 kJ mol^–1^, which must principally be associated with improved solvation of the free guest in water in its charged state, and the effect is larger (at least 16 kJ mol^–1^) for the diacid.

These sets of measurements represent promising examples of the use of a pH swing to control guest uptake and binding from cages; a swing of three orders of magnitude from 10^4^ to 10^1^ M^–1^ (*cf.* the behaviour of 1-aminoadamantane) would represent a change from 97% bound to 97% free for a 1 : 1 host–guest mixture at concentrations above 3 mM. An attractive feature of the system described here is that the cage **H** is remarkably stable with respect to pH. There is no sign of any decomposition between pH 2 and pH 12; the cage does slowly decompose over a period of hours at pH 12, but it is stable on the minute timescale required to record ^1^H NMR spectra.

Significantly, the guest 1-aminoadamantane is the prescription drug ‘amantadine’ which has been used to treat Parkinson's disease and as an anti-viral for treatment of influenza.^23^ Thus we have demonstrated pH dependent uptake and release of a drug molecule to/from the cage cavity, providing an interesting possible method of controlled drug release. Although release at pH 11 is not compatible with biological conditions, there are numerous other functional groups with p*K*_a_ values that fall in the physiological range, and we therefore investigated several representative examples.

**Fig. 6:**
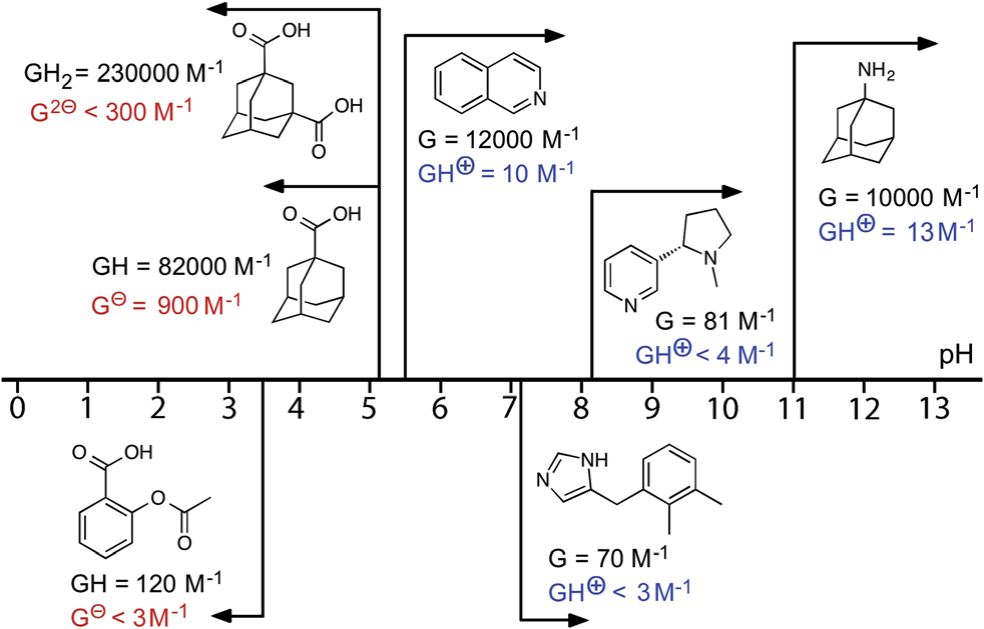
Graphical summary of association constants for guests in neutral and charged states (see also Table 1).

Starting from the family of bicyclic guests that we know can be accommodated in the cavity of the cage,^15,16^ we evaluated isoquinoline as a pH-dependent guest. Isoquinoline binds in slow exchange on the ^1^H NMR timescale [*K* = 1.2(5) × 10^4^ M^–1^]. The p*K*_a_ of isoquinoline is 5.5, and it was possible to measure the association constant of the protonated form at lower values of pH: *K* = 10(2) M^–1^, which is a change of three orders of magnitude in the association constant (ΔΔ*G* = 18 kJ mol^–1^). The behaviour of other molecules, some of which are of biological interest, as switchable guests is summarised in Fig. 6 (see also Table 1). Thus nicotine (p*K*_a_ = 8.1) binds with *K* = 81(20) M^–1^ in the neutral form, but when the tertiary amine group was protonated no evidence of binding was observed. The sedative and anaesthetic molecule detomidine^24^ has an imidazole moiety as the ionisable group (p*K*_a_ = 7.1), and the association constant drops from *K* = 70(30) M^–1^ for the neutral form to undetectably small following protonation. Binding of neutral aspirin (p*K*_a_ = 3.5) occurs with *K* = 120(30) M^–1^, and this also falls to undetectably small for the deprotonated anionic form. None of these examples matches the high binding affinity of the adamantane-based guests, presumably due to a less optimal shape/size match for the host cavity and less hydrophobic character, but they still show pH-induced switching of binding.

**Table 1:** Summary of binding constants and Δ*G*° values for formation of complexes of neutral and charged forms of the guests with the host cage **H** (water, 298 K)

Guest	p*K*_a_	Neutral form		Charged form	
		*K*/M^–1^	–Δ*G*°/kJ mol^–1^	*K*/M^–1^	–Δ*G*°/kJ mol^–1^
1-Amino-adamantane	10.9	1.0(3) × 10^4^	22.8(7)	13(7)	6(1)
1-Adamantane-carboxylic acid	5.1	8.0(2) × 10^4^	28.0(1)	9.0(5) × 10^2^	17.0(3)
1,3-Adamantane-dicarboxylic acid	4.8, 5.9	2.3(4) × 10^5^	30.6(4)	<300^*a*^ ^,^ ^*b*^	<14^*a*^ ^,^ ^*b*^
Isoquinoline	5.5	1.2(5) × 10^4^	23.3(8)	10(2)	5.7(5)
Detomidine	7.2	70(30)	10.5(8)	<3^*a*^	<3^*a*^
(–)-Nicotine	8.1	81(20)	10.9(5)	<4^*a*^	<3^*a*^
Aspirin	3.5	1.2(3) × 10^2^	11.9(6)	<3^*a*^	<3^*a*^

^*a*^In these cases, no evidence for guest binding was seen by ^1^H NMR spectroscopy at the concentrations used; upper limits for *K* (and hence Δ*G*) are estimated on the assumption that 5% of bound host is the minimum that could be detected.

^*b*^See footnote.‡

We note that for the two basic guests 1-aminoadamantane and isoquinoline for which association constants could be measured in both neutral and protonated forms, the value of ΔΔ*G* – *i.e.* the difference in free energy of binding between neutral and charged forms – is significantly larger (17 and 18 kJ mol^–1^, respectively) than with 1-adamantane-carboxylic acid (ΔΔ*G* = 11 kJ mol^–1^). This presumably reflects an additional electrostatic contribution to complex stability which depends on the charge of the guest. Thus we expect an attraction between **H** and adamantane-1-carboxylate which slightly stabilises the complex with the anionic guest and gives a smaller value of ΔΔ*G*, but a repulsion between **H** and protonated isoquinoline/protonated 1-aminoadamantane which slightly destabilises the complexes and gives a larger value of ΔΔ*G*. The consequence is a two order-of-magnitude swing for the pH-dependent binding constant of 1-adamantane-carboxylic acid but a three order-of-magnitude swing for 1-aminoadamantane and isoquinoline. Improved solvation of the charged form of the guest irrespective of sign is the dominant factor in determining ΔΔ*G*, but an additional electrostatic contribution is also evident.

**Fig. 7:**
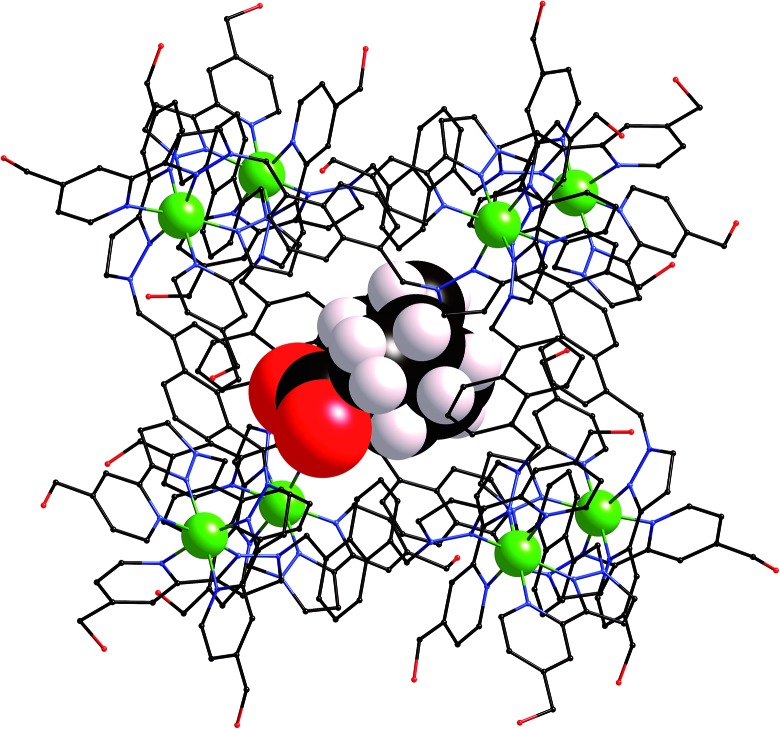
Structure of the **H**·(1-adamantane-carboxylic acid) complex from crystallographic data, showing the cage (in wireframe) and the encapsulated guest (space-filling mode).

**Fig. 8:**
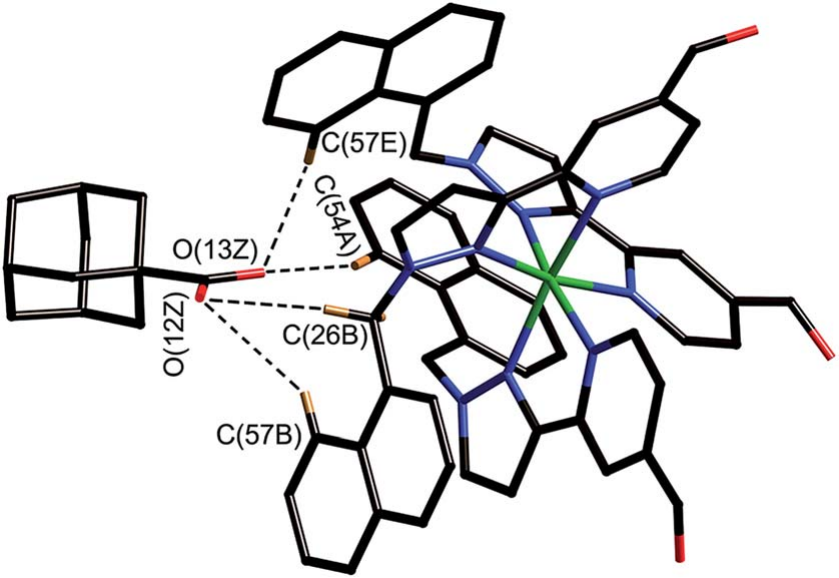
Close-up view from the crystal structure of the four closest contacts between the oxygen atoms of the guest and some of the naphthyl and methylene CH protons of the host in the binding pocket (see main text); the C···O distances lie in the range 2.68–2.86 Å.

We obtained a crystal structure of the complex of **H** with adamantane-1-carboxylic acid,§
§Crystallographic data for [Co_8_(L^w^)_12_](BF_4_)_16_·(C_11_H_16_O_2_): C_371_H_328_B_16_Co_8_F_64_N_72_O_26_, *M* = 8071.43 g mol^–1^, monoclinic, space group *C*2/*c*, *a* = 27.3936(7), *b* = 39.1227(10), *c* = 41.964(3) Å, *β* = 107.152(8)°, *U* = 42 973(4) Å^3^, *Z* = 4, *ρ*_calc_ = 1.248 g cm^–3^, *T* = 100(2) K, *λ* (Mo-Kα) = 0.71075 Å, *μ* = 0.393 mm^–1^. 147 295 reflections with 2*θ*_max_ = 55° were merged to give 49 124 independent reflections (*R*_int_ = 0.052). Final *R*_1_ [for data with *I* > 2*σ*(*I*)] = 0.163; w*R*_2_ (all data) = 0.469. The data collection was performed by the EPSRC National Crystallography Service at the University of Southampton (ref. 26). Data were corrected for absorption using empirical methods (SADABS) (ref. 27) based upon symmetry-equivalent reflections combined with measurements at different azimuthal angles. The structure was solved and refined using the SHELX suite of programs (ref. 28). The asymmetric unit contains one half of the molecule which lies astride an inversion centre. The asymmetric unit contains one half of the cage complex which lies astride an inversion centre, as well as one complete guest molecule whose atoms all have site occupancies of 0.5. Thus, the complete complex contains one guest molecule disordered over 2 symmetrically equivalent (and spatially overlapping) orientations with the O atoms pointing towards diagonally opposite corners Co(1) and Co(1A). The usual disorder of anions/solvent molecules and solvent loss characteristic of cage complexes of this type resulted in weak scattering, necessitating use of extensive geometric and displacement restraints to keep the refinement stable: these are described in detail in the CIF. We could locate and refine four of the expected eight [BF_4_]^–^ anions in the asymmetric unit; all show disorder of the F atoms. Large regions of diffuse electron density which could not be modelled, accounting for the remaining anions plus solvent molecules, were eliminated from the refinement using of the ‘SQUEEZE’ function in the PLATON software package (ref. 29). by immersing pre-formed crystals of **H** in a saturated solution of adamantane-1-carboxylic acid in *n*-hexane for 24 hours, resulting in uptake of the guest into the cavity of the host. This is a common method for incorporating guests reversibly into pre-formed hosts without loss of crystallinity.^25^ Given the fact that the guest was administered in its neutral acid form we assume that it is in this form in the host cavity, and not as the adamantane-1-carboxylate anion, which has a much lower binding affinity.¶
¶The distinction is not crystallographically obvious as extensive disorder of the tetrafluoroborate anions in the structure means that not all of them could be located – so we cannot use charge balance considerations to determine whether or not the guest is protonated. Although the two C–O bond distances of the carboxylic acid (or carboxylate) group appear to be approximately equivalent, *i.e.* there is no obvious short (double) and long (single) distinction between the C–O bonds, the presence of disorder of the entire guest over two orientations – plus the additional possibility of C=O/C–OH disorder within each orientation – means that we cannot draw any conclusion from the bond lengths. As is normal for cage complexes of this type, weak scattering resulted in a relatively high *R*_1_ value of 16%, which means that detailed analysis of structural minutiae is not appropriate, but the formation of the complex and its key structural features are clear (Fig. 7 and 8).

The adamantyl unit lies centrally in the cavity with the COOH group projected towards one of the two *fac* tris-chelate metal vertices which lie at either end of the long diagonal, with short CH···O contacts (2.7–2.9 Å) between the carboxylic acid oxygen atoms and some of the naphthyl and methylene protons on the interior surface of the host (shown by dotted lines in Fig. 8; the associated non-bonded O···C separations are in the range 3.5–3.8 Å). The guest is disordered over two symmetry-equivalent positions with 50% site occupancy in each: one orientation is shown in Fig. 7, and the alternative orientation (related by inversion) has the COOH group oriented towards the symmetry-equivalent opposite corner of the host. The two *fac* tris-chelate sites in **H** each provide a convergent group of CH protons in a region where the electrostatic potential on the internal surface is most positive, thus resulting in an H-bond donor pocket which is responsible for guest binding in organic solvents^15^ and which also provides an anchoring point for the polar part of the guest.^16*b*^

## Conclusions

We have demonstrated reversible pH-dependent uptake and release of several types of guest molecule into/out of the hydrophobic central cavity of a water-soluble coordination cage host; a graphical summary of the results is shown in Fig. 6. The largest swing is for 1-aminoadamantane for which the binding constant decreases from 10^4^ M^–1^ in the neutral form to 10^1^ M^–1^ in the protonated form. This change in binding affinity is driven principally by changes in solvation: the charged forms of the guest, regardless of whether they are cationic or anionic, bind more weakly than the neutral forms due to increased solvation of the free ion in water compared to the neutral form. Additional electrostatic interactions between (cationic) host and guest mean that the ΔΔ*G* values, and hence the efficacy of the pH swing at modulating guest uptake and release, are slightly larger between neutral/cationic guest pairs than between neutral/anionic guest pairs. The pH swing works over a range of values, from 3.5–11 depending on the p*K*_a_ of the guest, with several different functional groups (primary and tertiary amines, quinoline, imidazole, and carboxylic acid), and the cage is remarkably stable over this entire pH range. Some of the guests investigated (aspirin, amantadine, nicotine) have been used as drugs for which the ability to control uptake and release by an external perturbation is clearly a desirable target.
