# Supplementary material for: pH-dependent binding of guests in the cavity of a polyhedral coordination cage: reversible uptake and release of drug molecules
Source: Chem Sci. 2014 Jul 31;6(1):625–31. doi: 10.1039/c4sc02090a (PMC5588781; doi:10.1039/c4sc02090a)
Supplement: Supplementary file 1 [file SC-006-C4SC02090A-s001.pdf]

Electronic Supplementary Material (ESI) for Chemical Science.
This journal is © The Royal Society of Chemistry 2014

pH-Dependent binding of guests in the cavity of a polyhedral coordination cage: reversible uptake and release of drug molecules

William Cullen, Simon Turega, Christopher A. Hunter* and Michael D. Ward*

Supporting Information

---

1. Experimental details

The host cage **H** was prepared according to ref. 13a (main text). All pH measurements were made using a Hamilton Spintrode pH combination electrode at 298 K and calibrated with calibration standards at pH 4.01, 7.0 and 10.01. 400 MHz ^{1}H NMR spectra were recorded on a Bruker AV3-400 instrument.

For all host/guest binding experiments samples were prepared as follows. **Host solution:** the host cage (16 mg) was dissolved in 10 ml D_2O to make a 0.2 mM stock solution. **Guest solutions:** the guests were individually dissolved in 5 ml of the host solution (to keep the host concentration constant during the titration); the mass used varied with the desired concentration. All binding experiments were performed three separate times; the values for binding constants quoted are the average of the three measurements with the error quoted being two standard deviations from the mean.

pH titrations – guests in slow exchange

The guest solutions were equilibrated using a water bath thermostatted at 298 K. The pH was measured and then adjusted to the desired value by addition of NaOD or DCl (1 M). The ^{1}H NMR spectrum for each addition was measured.

The pKa was calculated by plotting pH against the ^{1}H NMR chemical shift of a selected guest signal; the resultant curve was fitted using the Microsoft Excel add-on 'Solver' by using a least squares fitting of the calculated curve to the data.

For a guest with a single pK_a:

$$\delta_{Calc} = \propto_0 \times \delta(G_0) + \propto_1 \times \delta(G_1)$$

Where δ is the chemical shift of the signal in question (ppm), $\delta(G_1)$ is the chemical shift of the charged species; and $\delta(G_0)$ is the chemical shift of the neutral species.
α_0 and α_1 describe the speciation of the neutral and mono-charged species respectively, and follow the relationship outlined below:

$$\propto_1 = \frac{10^{-pKa}}{10^{-pH} + 10^{-pKa}}$$

$$\propto_0 = 1 - \propto_1$$

For a guest with two pK_s (adamantane-1,3-dicarboxylic acid):

$$\propto_2 = \frac{(10^{-pKa1} \times 10^{-pKa2})}{(10^{-pH})^2 + (10^{-pKa1} \times 10^{-pKa2}) + (10^{-pKa1} \times 10^{-pH})}$$

$$\delta_{Calc} = \propto_0 \times \delta(A) + \propto_1 \times \delta(AH) + \propto_2 \times \delta(AH_2)$$

$$\propto_1 = \frac{10^{-pH} \times \propto_2}{10^{-pKa2}}$$

$$\propto_0 = 1 - \propto_1 - \propto_2$$

The binding constant at each pH point was calculated by measuring the integral of the free host and host-guest peaks relative to each other using Topspin's deconvolution feature. This was done for several pairs of peaks at each pH and the average value was taken.

pH titrations – fast exchange

The host and guest solutions were equilibrated using a water bath thermostatted at 298 K. The pH of both solutions was adjusted to the same desired value by the addition of NaOD or DCl (1 M). 12 samples were then prepared with varying host:guest ratios (varying from pure host to pure guest), with a total volume of 600 µl per sample. Measurements were performed using 8 inch NORELL 507-HP NMR tubes, sealed with pressure caps to ensure that no solvent loss occurred during the measurements. From the resulting ^1H NMR spectra, the concentration of guest was plotted against change in chemical shift (ppm) for a selected signal, and the resultant curve was fitted to a 1:1 binding isotherm to obtain a value for the binding constant. This was repeated with several signals from the same set of spectra and the value of *K* averaged.

Binding constants of the neutral and charged forms

In both cases (whether the guest was in fast or slow exchange) separate host/guest titrations were also performed at two fixed pH values – corresponding to the neutral and charged forms of the guest – to get the binding constants for the two extremes. In cases of weak binding for the charged guests (*e.g.* aspirin, detomidine, nicotine) where binding could not be detected in the NMR spectra, an upper limit for *K* has been estimated by assuming that formation of host/guest complex occurs to an extent of < 5% in these cases.

2. Additional data for NMR measurements

These figures follow the formats of Fig. 2 – 5 in the main text. In all cases the % cage occupied is the red curve; δ(ppm) for a selected ^{1}H signal is the blue curve.

(i) 1,3-Adamantane dicarboxylic acid:

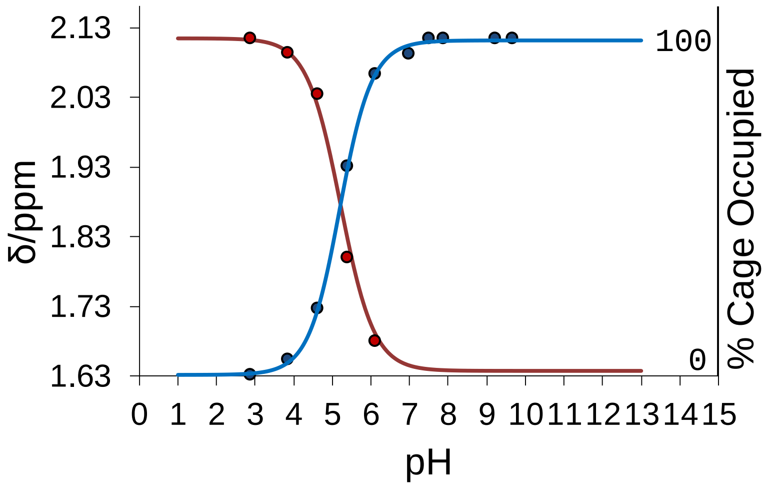

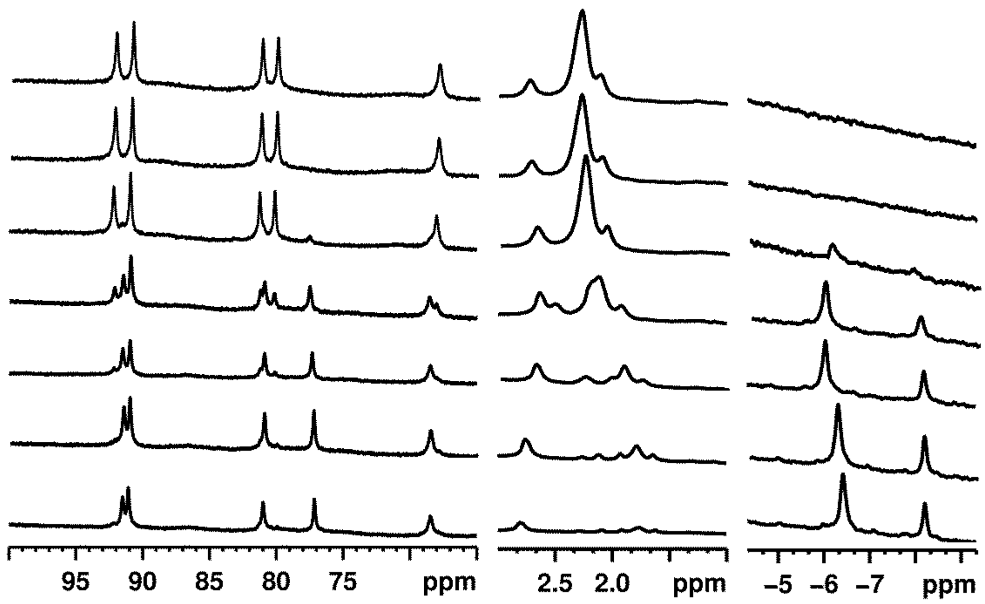

pH range bottom to top: 2.9, 3.85, 4.64, 5.4, 6.12, 6.99, 9.69.

(ii) Isoquinoline:

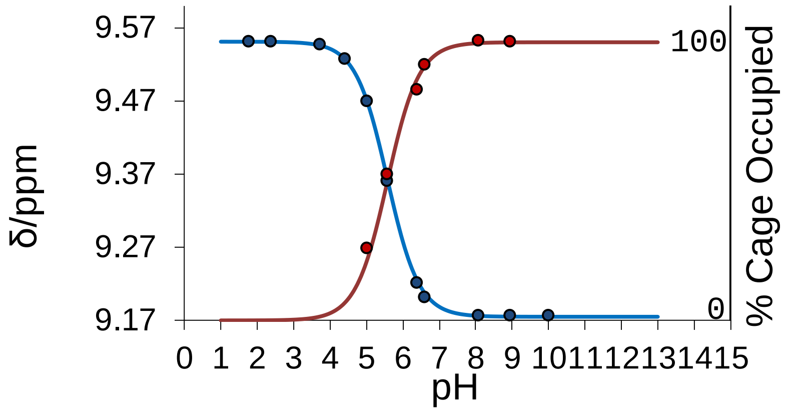

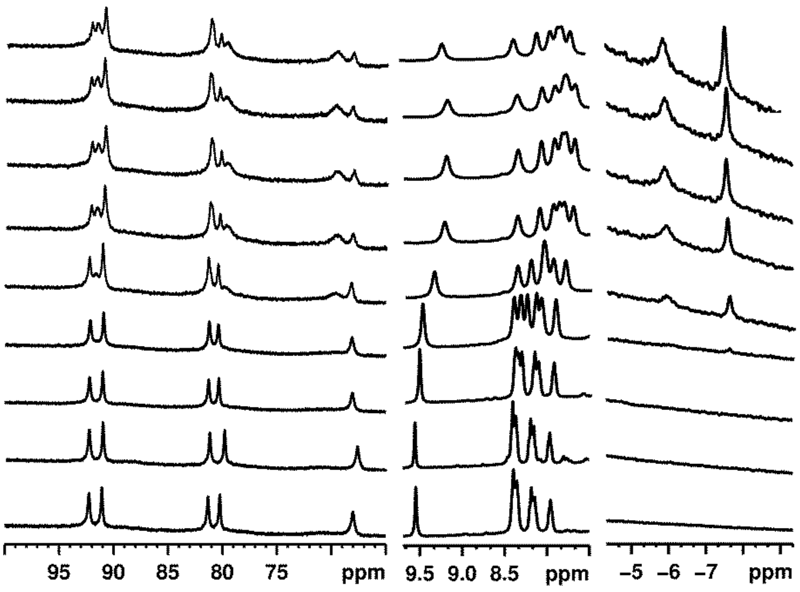

pH range bottom to top: 2.4, 3.73, 4.43, 5.04, 5.6, 6.62, 8.09, 8.96, 10.01.

(iii) Detomidine

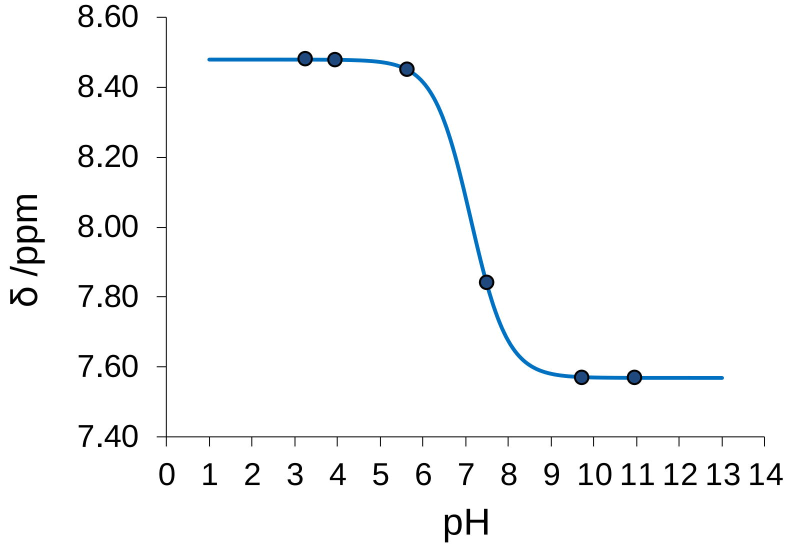

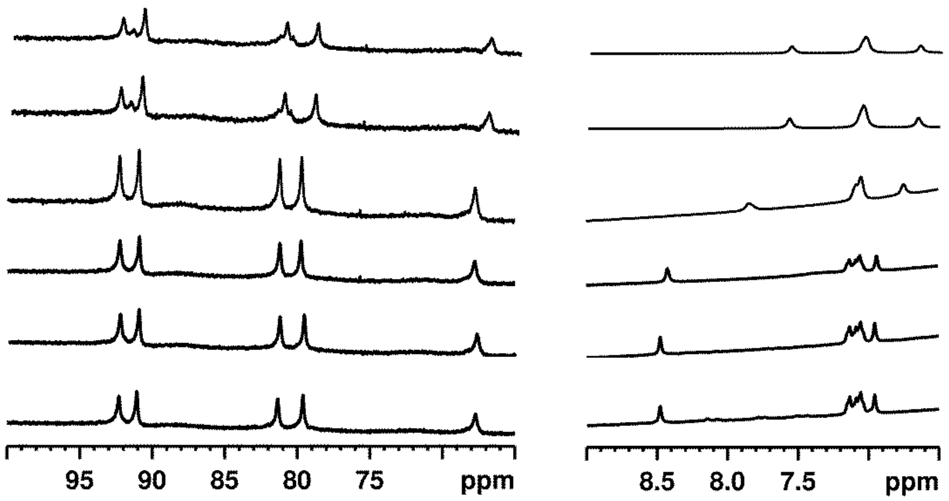

pH range bottom to top: 3.26, 3.94, 5.64, 7.5, 9.72, 10.95.

Due to solubility limits of detomidine during the pH titration, only the final 2 points could be integrated, so the cage occupancy is not shown on the graph above.

(iv) Nicotine (host/guest titration at a constant pH of 10.5, increasing guest concentration from bottom [0 mM] to top [6.3 mM], with [**H**] = 0.2 mM)

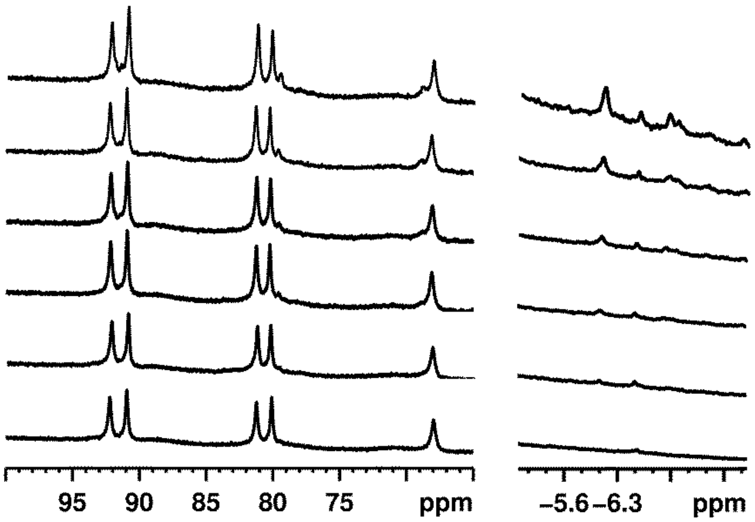

When this was repeated at pH 3.05 (cationic form of guest), no new signals associated with complex formation in slow exchange could be detected, as shown below:

Nicotine (host/guest titration at a constant pH of 3.05, increasing guest concentration from bottom [0 mM] to top [19 mM], with [**H**] = 0.2 mM)

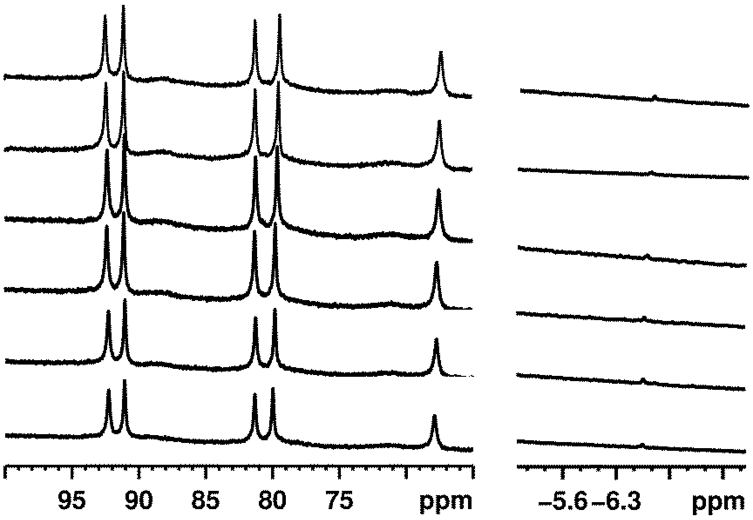

(v) Aspirin (host/guest titration at a constant pH of 1.6, with increasing guest concentration from bottom [0 mM] to top [17 mM], with [**H**] = 0.2 mM). The bottom trace shows the spectra overlaid, to illustrate the slight decrease in chemical shift for signals of of **H** as aspirin binds in fast exchange. Fitting the resulting binding curve gave the *K* value quoted in the main text

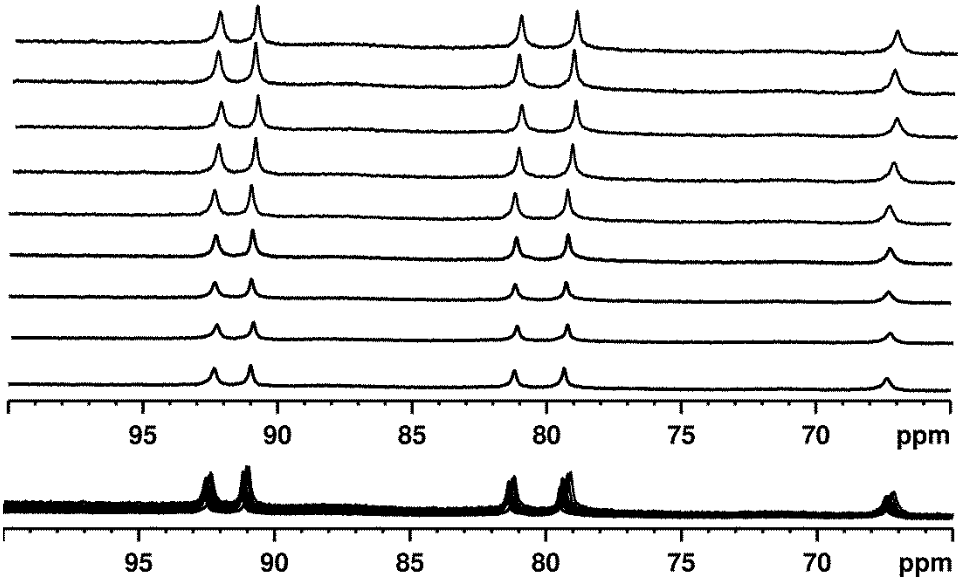

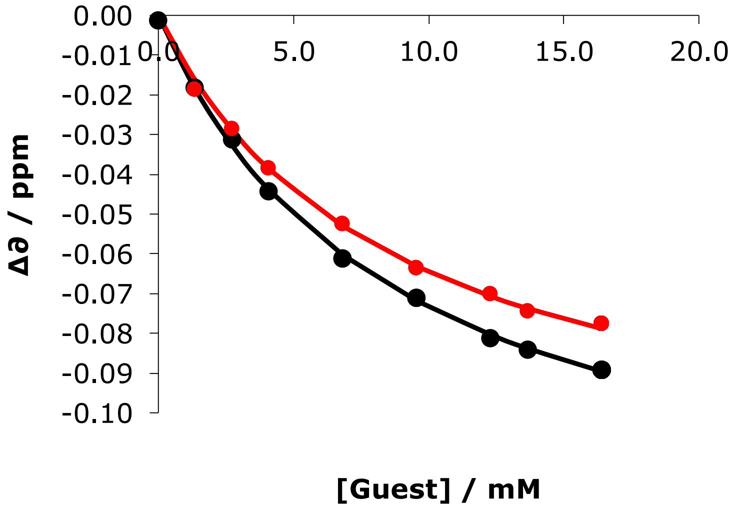

When this was repeated at pH 8.1 (anionic deprotonated form of guest) no change in the ^{1}H NMR chemical shifts of the guest were detected during the titration.
